# Exploring macrophage cell therapy on Diabetic Kidney Disease

**DOI:** 10.1111/jcmm.13983

**Published:** 2018-11-08

**Authors:** Roser Guiteras, Anna Sola, Maria Flaquer, Anna Manonelles, Georgina Hotter, Josep M. Cruzado

**Affiliations:** ^1^ Experimental Nephrology Department of Ciències Clíniques Universitat de Barcelona Institut d'Investigació Biomèdica de Bellvitge (IDIBELL) Hospitalet de Llobregat Barcelona Spain; ^2^ Networking Centre on Bioengineering Biomaterials and Nanomedicine (CIBER‐BBN) Barcelona Spain; ^3^ Hospital Universitari de Bellvitge Hospitalet de Llobregat Barcelona Spain; ^4^ Department of Ischemia and Inflammation Institut d'Investigacions Biomèdiques de Barcelona (IIBB) Barcelona Spain

**Keywords:** alternatively activated macrophages, cell therapy, plasticity

## Abstract

Alternatively activated macrophages (M2) have regenerative properties and shown promise as cell therapy in chronic kidney disease. However, M2 plasticity is one of the major hurdles to overcome. Our previous studies showed that genetically modified macrophages stabilized by neutrophil gelatinase‐associated lipocalin (NGAL) were able to preserve their M2 phenotype. Nowadays, little is known about M2 macrophage effects in diabetic kidney disease (DKD). The aim of the study was to investigate the therapeutic effect of both bone marrow‐derived M2 (BM‐фM2) and ф‐NGAL macrophages in the *db/db* mice. Seventeen‐week‐old mice with established DKD were divided into five treatment groups with their controls: D+BM‐фM2; D+ф‐BM; D+ф‐NGAL; D+ф‐RAW; D+SHAM and non‐diabetic (ND) (*db*/‐ and C57bl/6J) animals. We infused 1 × 10^6^ macrophages twice, at baseline and 2 weeks thereafter. BM‐фM2 did not show any therapeutic effect whereas ф‐NGAL significantly reduced albuminuria and renal fibrosis. The ф‐NGAL therapy increased the anti‐inflammatory IL‐10 and reduced some pro‐inflammatory cytokines, reduced the proportion of M1 glomerular macrophages and podocyte loss and was associated with a significant decrease of renal TGF‐β1. Overall, our study provides evidence that ф‐NGAL macrophage cell therapy has a therapeutic effect on DKD probably by modulation of the renal inflammatory response caused by the diabetic milieu.

## 1 INTRODUCTION

Chronic kidney disease (CKD) has been recognized as a major health problem worldwide^1^ and there is still a global rising incidence and prevalence.^2^ Diabetic kidney disease (DKD) is the leading cause of CKD in developed countries.^3^ Diabetes involves activation of chronic inflammation and immune response.^4^ Therefore, the progression of DKD is associated with increased of pro‐inflammatory cytokines such as tumour necrosis factor‐α (TNF‐α) and significant inflammatory cell infiltration of the kidney. Finally, the advanced form of DKD displays prominent transforming growth factor (TGF‐β) up‐regulation, mesangial expansion and glomerulosclerosis.^5^ Indeed, TGF‐β1 increases extracellular matrix accumulation through the stimulation of collagen IV and fibronectin production,^6^ resulting in interstitial fibrosis and glomerular sclerosis.

Some drug therapies targeting inflammation on DKD have been explored with variable success.^7^ On the other hand, the potential role of endogenous bone marrow‐derived and stem cells in the repair of kidney damage is still under exhaustive investigation.^8^ Therefore, interventions such as cell‐based therapy are being broadly developed aimed to prevent chronic disease progression^9^ although very few may induce renal repair.^10^ Among potential therapeutic strategies, macrophage cell‐based therapy provides discordant results.^11^, ^12^ Macrophages are associated with tissue damage although they have also a critical role in host defense and tissue repair.^13^, ^14^ Alternatively Activated Macrophages (AAM) also called as M2, have anti‐inflammatory functions and express high levels of mannose receptor (CD206), arginase and IL‐10.^15^, ^16^ Lipocalin‐2 (Lcn‐2), also called Neutrophil Gelatinase‐Associated lipocalin (NGAL), is able to promote M2 polarization.^17^, ^18^ M2 may help to overcome inflammation and through high endocytic clearance capacities mediate wound healing, tissue remodelling and repair.^14^ Nevertheless, M2 macrophages are also associated with fibrosis as they can secrete components of the extracellular matrix and produce growth factors that activate epithelial cells and fibroblasts, including TGF‐β.^19^ M2 macrophages play a controversial role in DKD but studies are scarce.^20^ Hence, modulating macrophages to a protective phenotype to reduce kidney injury in chronic kidney disease still needs further investigation. Nevertheless, the mechanisms regulating the distinct functions of macrophages during tissue repair remain largely unclear.^21^

We have previously demonstrated that anti‐inflammatory effects induced by hepatocyte growth factor (HGF) gene therapy enhanced the presence of bone marrow‐derived M2 (BM‐ΦM2) macrophages in the glomeruli of diabetic mice and halted DKD progression. Therefore, in this study we sought to investigate whether infusion of modulated ex‐vivo BM‐ΦM2 macrophages provide a therapeutic effect on DKD in obese *db/db* mice. Our previous results in UUO mice demonstrated that those BM‐ΦM2 macrophages secrete TGF‐β, did not diminish inflammation and displayed a phenotypic M1 switch when they faced a pro‐inflammatory and pro‐fibrotic milieu.^22^ To overcome this limitation, we also investigate an alternative macrophage therapy transduced with Neutrophil gelatinase‐associated lipocalin (NGAL), which is a M2 phenotypically stabilized cell line overexpressing the anti‐inflammatory cytokine IL‐10 and showing low TGF‐β secretion.^23^

## 2 MATERIAL AND METHODS

### 2.1 Animals

The experiments complied with the current legislation on animal experiments in the European Union, and the principles of laboratory animal care were approved by our institution's Ethics Committee for Animal Research. Ten‐week‐old diabetic female C57BL/Ks/J mice (*db/db*) and non‐diabetic (*db*/‐ and C57bl/6J) female mice were purchased from Janvier (Laval, France). Animals were given free access to water and a standard laboratory chow diet.

### 2.2 Macrophage cell culture

#### 2.2.1 Φ‐BM and BM‐ΦM2 macrophages

Primary cultures of murine macrophages were obtained from bone marrow of C57bl/6J mice purified by CD11b+ negative selection (EasySep, Grenoble, France). Bone marrow‐derived monocytes were matured in DMEM/F12 + GlutaMAX (Gibco, Waltham, MA, USA) medium supplemented with 10% FBS, 1% P/S and with 20 ng/mL macrophage colony stimulating factor (M‐CSF) to become macrophages (Φ‐BM) (ProSpec, East Brunswick, NJ, USA). For BM‐ΦM2, 10 ng/mL of IL‐4 and IL‐13 (Invitrogen, Carlsbad,, CA, USA) were added the last 3 days to become M2 macrophages. No IL‐4 and IL‐13 cytokines were added in Φ‐BM macrophage culture. Thus, Φ‐BM was used as a control for BM‐ΦM2 macrophages.

#### 2.2.2 Φ‐RAW and Φ‐NGAL macrophages

Mice RAW 264.7 macrophages (Φ‐RAW) were cultured in DMEM/F12 + GlutaMAX medium supplemented with 10% FBS and 1% P/S. For Φ‐NGAL group, RAW 264.7 macrophages were cultured and then transduced ex vivo with the adenoviral vector NGAL as previously described by the group.^24^ The adenoviral vector carrying cDNA encoding recombinant NGAL was elaborated, amplified and purified by ViraQuest, Inc (North Liberty, IA, USA). We previously reported the control vector Φ‐βGAL as a control vector for the Φ‐NGAL transduction.^22^ We analysed Φ‐βGAL macrophages before infusion and showed no differences compared to Φ‐RAW macrophages (Figure S1), but obviously show a difference regarding their average life expectancy. Tacking this feature into account, we use NGAL modified cell line for chronic models, such as DKD or Unilateral Ureteral Obstruction (UUO).

### 2.3 Experimental design and study groups

Mice were divided into five treatment groups with their respective controls and followed for four weeks: (a) D+BM‐ΦM2 (n = 9), diabetic animals treated with BM‐ΦM2; (b) D+Φ‐BM (n = 9), diabetic animals treated with Φ‐BM as control for BM‐ΦM2; (c) D+Φ‐NGAL (n = 9), diabetic animals treated with Φ‐NGAL; (d) D+Φ‐RAW (n = 9), diabetic animals treated with Φ‐RAW as control for Φ‐NGAL; (e) D+SHAM (n = 9), diabetic animals treated with saline Buffer as age‐matched control group and (f) *db*/‐ and C57bl/6J mice as non‐diabetic (ND) (n = 8) animals. For cell therapy, one million (1 × 10^6^) of Φ‐BM, BM‐ΦM2, Φ‐RAW or Φ‐NGAL macrophages were infused on 17‐week‐old and 19‐week‐old *db/db* mice, respectively, by intravenous injection in the tail vein. Mice were killed under anaesthesia and evaluated on 21‐week‐old.

### 2.4 Monitoring

Animals were followed from 10 to 21 weeks of age. During this period glucose levels and body weight were measured weekly. Glucose was measured using the ACCU‐CHEK Performa blood glucose meter (Roche, Basel, Switzerland). Blood was obtained from the tail vein and samples were collected in order to analyse the serum creatinine at three time points: before cell therapies (15 weeks), when cell therapies were performed (20 weeks) and before mice were killed (21 weeks). Mice were placed in metabolic cages in order to collect 24 hours urine specimens before cell therapies (15 weeks), during cell therapies (18 weeks, 19 weeks and 20 weeks, respectively) and after macrophage cell therapies (21 weeks). Urine samples were collected in order to analyse the urinary albumin concentration. Urine creatinine and serum creatinine were determined following Jaffe's and GLDH reactions (Olympus Autoanalyzer AU400, Hamburg, Germany) in the Veterinary Clinical Biochemistry Laboratory of Universitat Autònoma de Barcelona. Urine albumin excretion was determined using a specific commercially available ELISA kit (Albumine Blue, la Hulpe, Belgium).

### 2.5 Detection of the infused macrophages in the kidney of *db/db* mice

In order to assess whether adaptive transfer of exogenous ф‐NGAL macrophages reached diabetic kidneys, macrophages were cultured and then stained with a red fluorescent membrane label Vivotrack680 (Vertex, Barcelona, Spain) according to manufacturer's instruction. One million (1 × 10^6^) of labelled macrophages were injected into mice by a single tail‐vein injection. Mice were killed 24 hours after macrophage injection and were assayed and quantified. Data were acquired and analyzed using FACS Diva software.

### 2.6 Optical microscopy, immunohistochemical, immunofluorescence and confocal studies

For conventional histology, 3 μm thick renal sections were fixed in 10% (vol./vol.) formalin and embedded in paraffin. Renal slices were stained with fibronectin and Masson's trichrome for optical microscopy assessment. Fibronectin (1:500) (Abcam, Madrid, Spain) and Anti‐collagen IV (1:100; Millipore, Livingston, UK) were stained using the immunohistochemical technique described previously.^25^ Slides were analysed by optical microscopy to assess fibrosis and glomerulosclerosis and quantified using ImageJ software in each non‐overlapping cortical field from the cortical region. For histological analysis 21‐35 glomeruli per animal were evaluated. A magnification of ×400 was assessed to quantify histological sections (7 per animal). Values are obtained as relative stained area (%).

Confocal studies (Leica TCS‐SL, Mannheim, Germany) were performed on paraffin‐embedded renal tissue. Double immunolabelling of antimouse F4/80 (1:50, LabClinics, Barcelona, Spain) and anti‐CD86 (1:50, Abcam) were analysed using immunofluorescence. Slides were stained with F4/80 for macrophages and CD86 for the M1 subpopulation. Macrophages and M1 subpopulation were evaluated in ten glomeruli per slide and per animal in a blinded manner (n = 9 each group). The percentage of M1+ with respect to F4/80+ macrophages was calculated. Alexa Fluor 488 (green) and Alexa Fluor555 (red) were used as secondary antibodies (Invitrogen). For podocyte assessment, nuclear marker WT‐1 (1:30 Santa Cruz Biotechnology, Heidelberg, Germany) and Alexa Fluor 488 as a secondary antibody (green) (1:1000, Invitrogen) were used. In a blinded manner, podocytes were quantified in ten glomeruli per sample and calculated as podocyte/total nuclei ratio. Nuclei were stained blue with DRAQ5.

### 2.7 Quantitative real‐time PCR

RNA was extracted from kidney as previously described.^25^ A total amount of 400 ng RNA was used to perform the reverse transcription using a High‐Capacity cDNA Reverse Transcription Kit (Applied Biosystems, Warrington, UK). TGF‐β1, Megalin, IL‐10, TNF‐α, Mannose receptor, IL‐1β and CD40 were quantified by TaqMan real‐time PCR (ABI, Prism 7700, Applied Biosystems) using the comparative 2^−[delta][delta]Ct^ method.

### 2.8 Cytokine analysis

Serum cytokines were quantitatively measured by FACS Canto with the mouse inflammation kit cytometric bead array (CBA) from BD Bioscience (San Jose, CA, USA). Data were acquired and analysed using BD CBA software.

### 2.9 ELISA

Frozen kidney samples were dissolved and homogenized into a specific buffer and centrifuged at 3000 *g* for 15 minutes at 4°C. The protein concentration was analysed with a Bradford Assay kit (Thermo Scientific, Rockford, IL, USA). Quantitative assessment of TNF‐ α (DY410) and TGF‐β1 (DY1679) proteins were carried out by enzyme‐linked immunosorbent assay (ELISA) (Duo Set ELISA, R&D, Minneapolis, MN, USA).

### 2.10 Statistical analysis

All data are presented as mean ± SE. Group means were compared with either the Student's *t*‐test or ANOVA for parametric values, or the Mann–Whitney *U* test or Krustal‐Wallis test for non‐parametric values. *P* ≤ 0.05 was considered to be statistically significant. All statistical analyses were carried out using StatView software.

## 3 RESULTS

### 3.1 BM‐ΦM2 cell therapy does not have therapeutic effect on DKD

**Figure 1:**
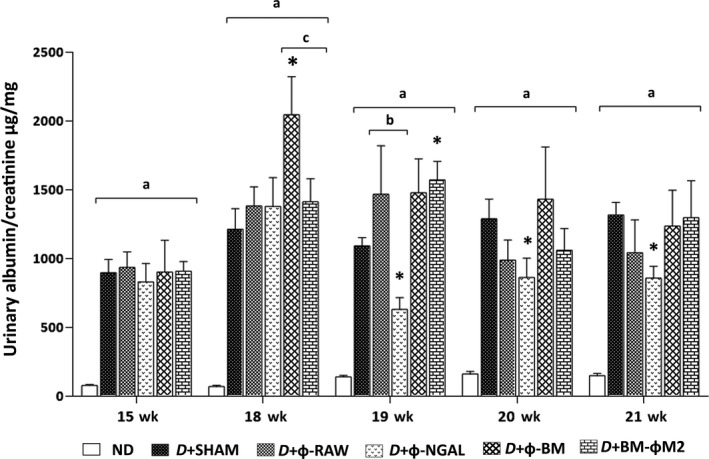
Albumin evolution analysis. Urinary albumin and creatinine were assessed before (15 wk) and during (18 wk, 19 wk, 20 wk and 21 wk) macrophage cell therapies. Non‐diabetic (ND) mice did not show an increase of the albumin/creatinine ratio. Control diabetic mice (D+SHAM) displayed persistent albumin/creatinine ratio whereas D+Φ‐NGAL treated mice showed a significant reduction from 19 wk. D+Φ‐RAW as a control to D+Φ‐NGAL and D+Φ‐BM as a control to D+BM‐ΦM2 did not show a significant decrease in any time‐point. Any other group exhibited a significant decrease. All diabetic mice (D) showed statistical significance vs ND animals. Data are represented as mean ± SE. *P ≤ 0.05 vs D+SHAM; ^a^ *P* ≤ 0.05 vs ND; ^b^ *P* ≤ 0.05 vs D+Φ‐RAW; ^c^ *P* ≤ 0.05 vs D+Φ‐BM

**Figure 2:**
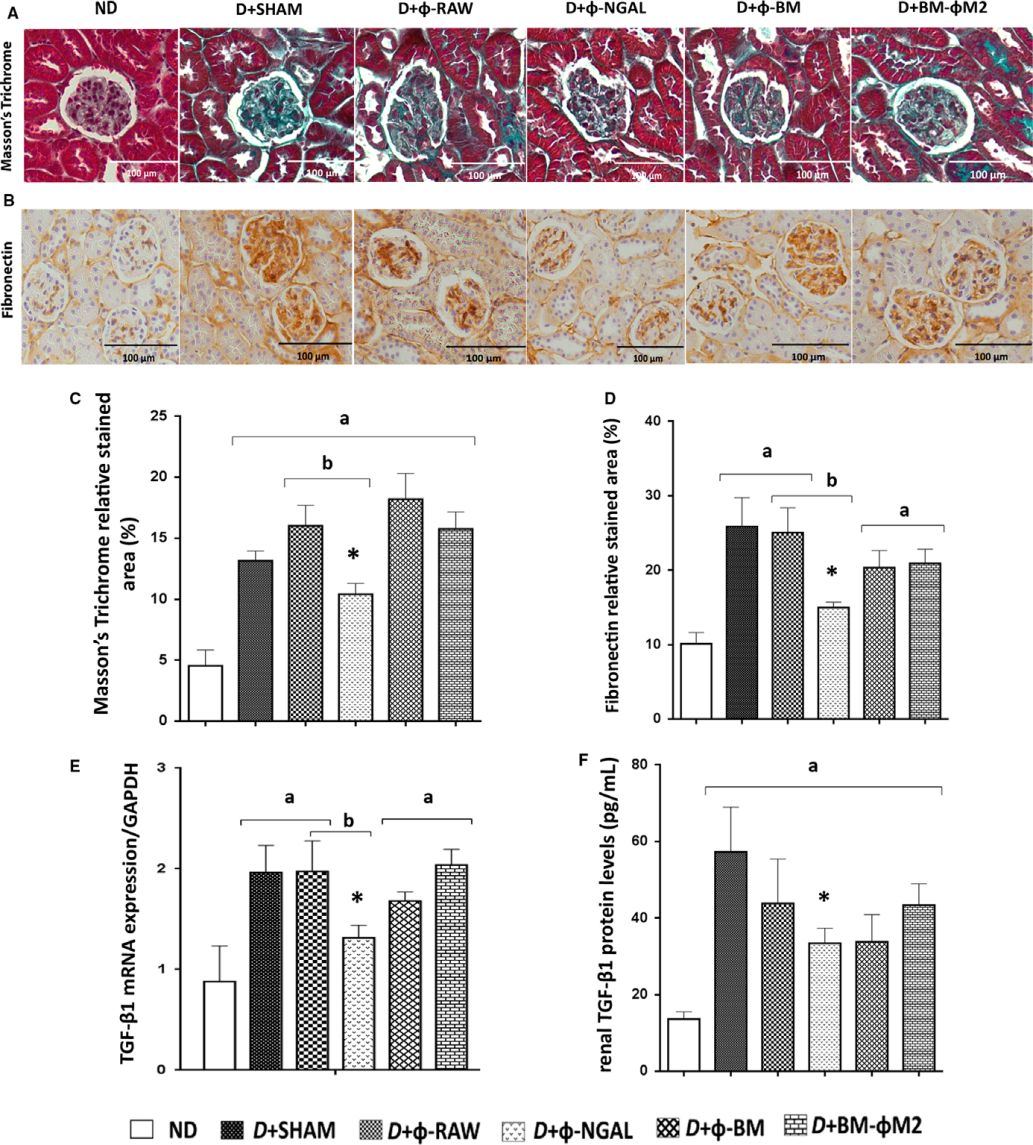
Effect of different macrophage therapies on glomerulosclerosis and fibronectin expression in kidney. Representative histological images of (A) Masson's trichrome and (B) fibronectin analysis in non‐diabetic (ND) and diabetic (D) treated groups. Control diabetic mice (D+SHAM) showed increased glomerulosclerosis and fibronectin, which was improved by Φ‐NGAL macrophage infusion as demonstrated by quantification in ImageJ software (C, D). (E) mRNA gene expression of kidney tissue revealed a significant decrease of TGF‐β1 in diabetic D+Φ‐NGAL treated mice for its control D+Φ‐RAW and for the diabetic control D+SHAM. (F) Renal tissue homogenates from treated and non‐treated mice were prepared for enzyme‐linked immunosorbent assay (ELISA) to determine protein levels of TGF‐β1 displaying a significant decrease only in D+Φ‐NGAL mice compared to diabetic control D+SHAM. Data are represented as mean ± SE. *P ≤ 0.05 vs D+SHAM; ^a^ *P* ≤ 0.05 vs ND; ^b^ *P* ≤ 0.05 vs D+Φ‐RAW; ^c^ *P* ≤ 0.05 vs D+Φ‐BM

**Figure 3:**
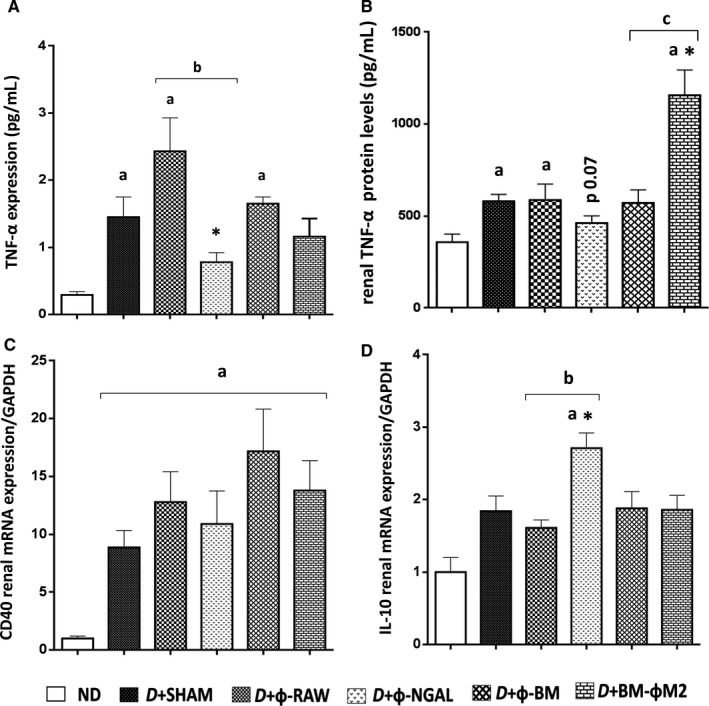
Φ‐NGAL macrophage therapy reduces inflammation. A, Serum levels of the pro‐inflammatory TNF‐α cytokine was assessed and showed a significant decreased expression in D+Φ‐NGAL treated mice compared with D+SHAM and its control group D+Φ‐RAW. B, Quantification of TNF‐α protein levels from kidney tissue measured by ELISA almost reached statistical significance in D+Φ‐NGAL treated mice. Its control group D+Φ‐RAW showed the same expression as diabetic control mice (D+SHAM). C, CD40 mRNA expression levels demonstrated a decrease of this molecule although it did not reach statistical significance. D, The anti‐inflammatory cytokine IL‐10 displayed a significant increase in D+Φ‐NGAL group compared both with non‐diabetic (ND), D+SHAM and D+Φ‐RAW. Data are represented as mean ± SE. *P ≤ 0.05 vs D+SHAM; ^a^ *P* ≤ 0.05 vs ND; ^b^ *P* ≤ 0.05 vs D+Φ‐RAW; ^c^ *P* ≤ 0.05 vs D+Φ‐BM

**Figure 4:**
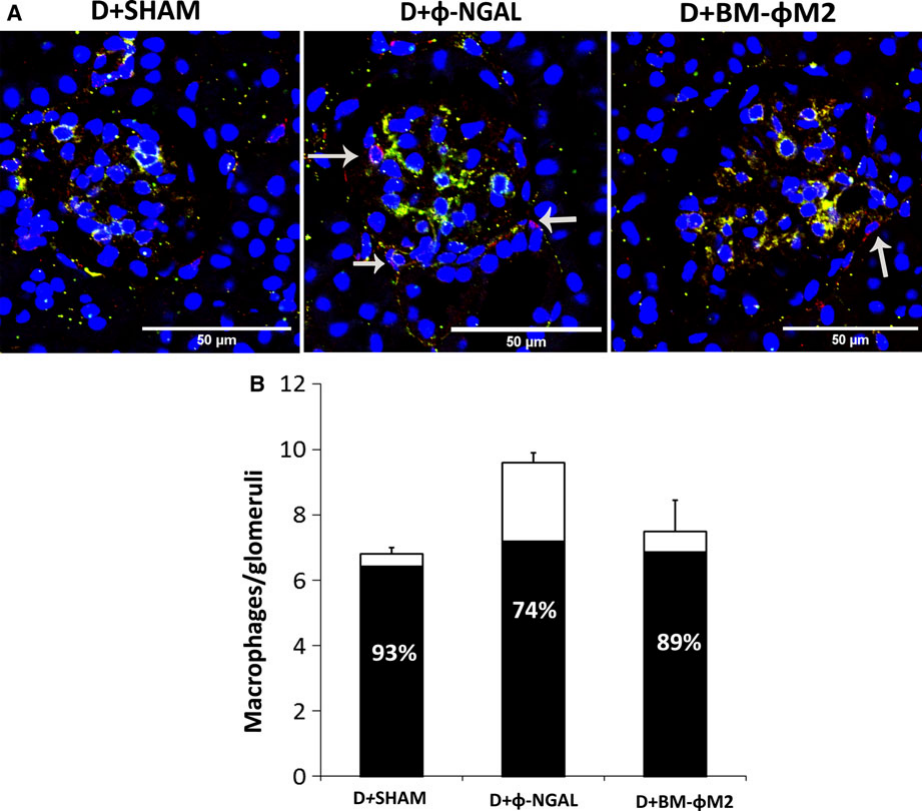
Evaluation of glomerular macrophages expressing CD86 (M1) marker. Glomerular detail (×400) using F4/80 (red) and CD86 (green) staining in order to identify general macrophages and M1 phenotype subpopulation (A). Double positive macrophages become yellow. The arrows show F4/80 positive macrophages that do not co‐express CD86 marker. (B) Number of macrophages per glomeruli. Each bar represents the mean number of macrophages F4/80 per glomeruli and per group. The black area in the bars indicates the percentage of the total macrophages that show M1 phenotype (n = 9 per group). Data are represented as mean ± SE

**Figure 5:**
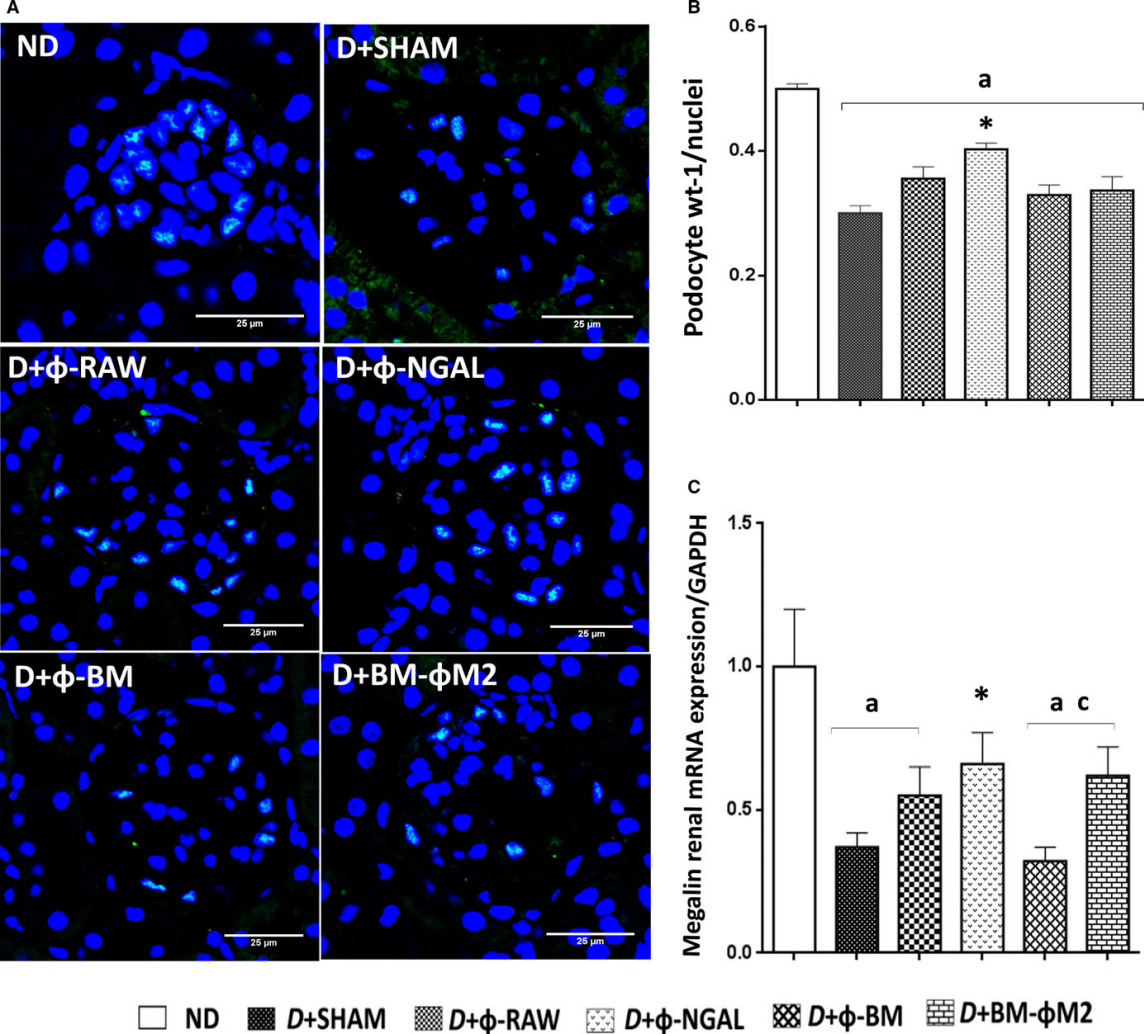
Evaluation of glomerular podocytes. Podocytes are stained with WT‐1 (green) and nuclei with DRAQ5 (blue) (×400). B, Quantification number of podocytes per nuclei in ten glomeruli; ф‐NGAL therapy increased the presence of glomerular podocytes while its control group ф‐RAW and the others showed similar results. C, Megalin mRNA expression in kidney tissue demonstrated more renal integrity in D+ф‐NGAL mice. Each bar represents the mean ± SE from each group. n = 9 in each group diabetic group and n = 8 in ND group. *P ≤ 0.05 vs D+SHAM; ^a^ *P* ≤ 0.05 vs ND; ^b^ *P* ≤ 0.05 vs D+Φ‐RAW; ^c^ *P* ≤ 0.05 vs D+Φ‐BM

All experimental diabetic groups (D) showed significantly higher body weight, higher albuminuria and diuresis than the non‐diabetic group (ND) (Table 1, Figure 1). There were no significant differences between D+BM‐ΦM2 and D+SHAM mice regarding albuminuria (Table 1 and Figure 1), glomerulosclerosis (Figure 2A‐D), renal mRNA gene expression (Figures 2E and 3C, D), glomerular pro‐inflammatory macrophage phenotype (Figure 4) and loss of podocytes (Figure 5A, B).

**Table 1:** Baseline and after treatment variables associated with diabetic kidney disease

Characteristic	Non‐diabetic	D+SHAM	D+ф‐RAW	D+ф‐NGAL	D+ф‐BM	D+BM‐ф‐M2
*Week 16 (pre‐treatment)*						
Body weight (g)	17.93 ± 1.2	43.05 ± 2.7^a^	42.32 ± 5.5^a^	45.13 ± 3.6^a^	39.56 ± 2.5^a^	42.73 ± 3.8^a^
Glycaemia (mg/dL)	143.5 ± 18.1	552.1 ± 54.7^a^	583.6 ± 54.7^a^	534.4 ± 40.1^a^	599 ± 20.4^a^	590.75 ± 17.1^a^
Albuminuria (μg/24 h)	17.24 ± 1.3	420.53 ± 3.6^a^	422.97 ± 164.4^a^	403.4 ± 150^a^	413.7 ± 106.2^a^	441.1 ± 115.3^a^
Diuresis (mL)	0.25 ± 0.1	9.2 ± 3.3^a^	12.3 ± 3^a^	9.9 ± 1.1^a^	8.4 ± 3.2^a^	11.6 ± 4^a^
*Week 20 (post‐treatment)*						
Body weight (g)	22.83 ± 1.3	40.77 ± 2.5^a^	38.71 ± 5.7^a^	40.56 ± 4.5^a^	34.35 ± 1.7^a^	41.04 ± 5.1^a^
Glycaemia (mg/dL)	131.4 ± 14.1	572.7 ± 43.4^a^	569 ± 35.8^a^	583.2 ± 20.9^a^	563.2 ± 53.4^a^	550.22 ± 59.9^a^
Albuminuria (μg/24 h)	39.19 ± 17.5	881.1 ± 140^a^	783.91 ± 210.1^a^	621.97 ± 90.4^a^^,^ b	1067.7 ± 357.1^a^	893.57 ± 187.3^a^
Diuresis (mL)	1.15 ± 0.3	21.7 ± 4.2^a^	24.6 ± 7.1^a^	22.9 ± 4.2^a^	19.3 ± 1.15^a^	21.2 ± 4.2^a^

Diabetic mice (D) showed a significant increase of body weight, glycaemia, albuminuria and diuresis compared to non‐diabetic (ND), both pre and post‐treatment. After treatment, D+ф‐NGAL displayed a reduction of the albumin excretion. Data are represented in mean ± SE.

^a^ *P* ≤ 0.05 vs ND; ^b^ *P* ≤ 0.05 vs D+SHAM.

### 3.2 Φ‐NGAL macrophage cell therapy displays a therapeutic effect on DKD

Treatment with Φ‐NGAL macrophages resulted in significant reduction in urinary albumin from the 19‐week compared to the diabetic control mice (D+SHAM) (Table 1, Figure 1). Furthermore, the proper Φ‐NGAL control group, the D+Φ‐RAW group, did not show any therapeutic effect on DKD. Moreover, those diabetic animals receiving Φ‐NGAL treatment were the only group displaying a significant reduction in glomerulosclerosis as well as collagen IV and fibronectin glomerular deposition compared to D+SHAM and its control group D+Φ‐RAW mice (Figure 2A‐D).

Infused macrophages reached diabetic kidneys as shown in Figure S2. Representative plots display that 7.6% of kidney cells were detected as Vivotrack positive cells. Φ‐NGAL macrophages were stable M2‐like in terms of anti‐inflammatory and pro‐reparative profile. We measured IL‐10, Mannose Receptor (MR), TNF‐α and IL‐1β production in transduced macrophages (Figure S3). Results showed an up‐regulation of anti‐inflammatory mediators in Φ‐NGAL, even when stimulated with the pro‐inflammatory mediator LPS.

### 3.3 Φ‐NGAL macrophage cell therapy reduces kidney TGF‐β1 overexpression in DKD

As showed in Figure 2E and in agreement with histological data, Φ‐NGAL treated mice showed a reduction in the TGF‐β1 mRNA expression in kidney tissue. Kidney TGF‐β1 mRNA was significantly increased in diabetic mice (D+SHAM) compared to ND. Kidney TGF‐β1 mRNA remained high in the D+Φ‐BM, D+BM‐ΦM2 and D+Φ‐RAW groups (Figure 2E). Moreover, kidney TGF‐β1 protein expression was measured by ELISA and was also significantly reduced in the D+Φ‐NGAL group (Figure 2F).

### 3.4 Φ‐NGAL cell therapy reduces inflammation in DKD

We first measured serum levels of some pro‐inflammatory cytokines in all groups in order to test whether macrophage infusion could modulate inflammation in DKD. The D+SHAM, D+Φ‐RAW and D+Φ‐BM groups showed higher TNF‐α levels than ND group (Figure 3A). On the contrary, TNF‐α levels were significantly lower in D+Φ‐NGAL treated animals compared with diabetic non‐treated D+SHAM and their control D+Φ‐RAW (Figure 3A). Of note, no major differences were observed between the D+BM‐ΦM2 group and its control, the D+Φ‐BM group. We also evaluated the kidney tissue TNF‐α protein levels by ELISA (Figure 3B). Only the D+Φ‐NGAL group showed a reduction in TNF‐α kidney protein. Its control group, D+Φ‐RAW, showed similar TNF‐α than D+SHAM controls. Although serum TNF‐α was similar in the D+BM‐ΦM2 and D+SHAM groups, kidney TNF‐α protein expression, assessed by ELISA, was significantly higher in the D+BM‐ΦM2 than in the D+SHAM and showed statistical significance compared to its control group D+Φ‐BM (Figure 3B).

The kidney mRNA CD40 gene expression was up‐regulated in diabetic animals and it was marginally reduced after D+Φ‐NGAL cell therapy (Figure 3C). Interestingly, the anti‐inflammatory IL‐10 kidney gene expression was significantly increased in the D+Φ‐NGAL group compared with both diabetic control (D*+*SHAM) and non‐diabetic groups (ND) (Figure 3D).

### 3.5 Φ‐NGAL macrophage cell therapy reduces the glomerular M1 macrophage ratio

We evaluated the expression of M1 phenotype in glomerular macrophages by confocal microscopy. Macrophages were mainly localized around the glomeruli (Figure 4A). As shown in Figure 4B, diabetic control mice (D+SHAM) displayed a high ratio of CD86+/F4/80+ macrophages, achieving almost 100%. Φ‐NGAL macrophage infusion was associated with increased number of glomerular macrophages, although the proportion of CD86+ (M1) decreased to 74%. On the other hand, D+BM‐ΦM2 treated mice showed similar proportion of CD86+/F4/80 glomerular macrophages to diabetic control mice (D+SHAM).

### 3.6 Φ‐NGAL macrophage cell therapy abrogates podocyte loss and preserves kidney integrity in DKD

We analysed whether macrophage infusion modified the characteristic podocyte loss that appears in DKD. Thus, diabetic control mice (D+SHAM) displayed a reduced number of podocytes compared to the non‐diabetic (ND) mice (Figure 5A, B). Remarkably, D+SHAM treated group showed a decreased podocyte number compared to D+Φ‐NGAL group. In fact, podocyte number in the Φ‐NGAL group was similar to that in ND group. Φ‐BM and BM‐ΦM2 treated mice showed no differences compared to D+SHAM. Moreover, megalin mRNA gene expression in kidney tissue examination revealed that epithelial integrity was only preserved in D+Φ‐NGAL‐treated mice (Figure 5C).

### 3.7 Φ‐βGAL vector transduction did not show alternative M2 phenotype

**Figure 6:**
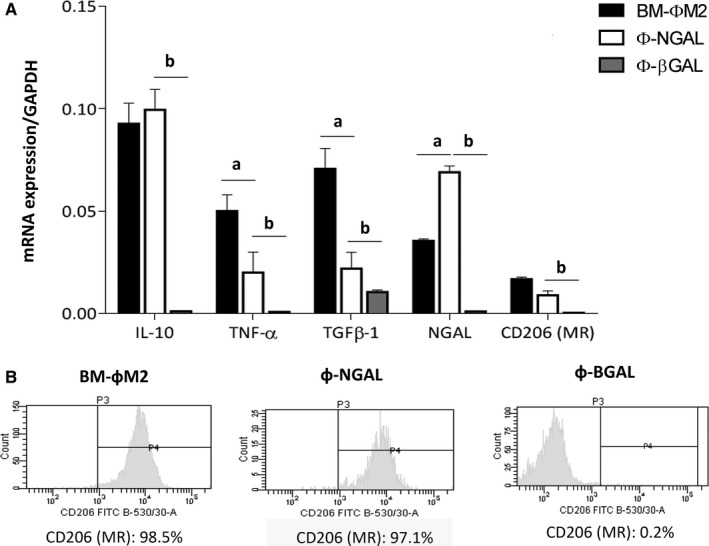
Macrophage gene expression profile and phenotype assessment before infusion. A, Macrophages were cultured and mRNA expression of IL‐10, TNF‐α, TGFβ‐1, NGAL and CD206 were measured by qPCR in fresh cultured BM‐фM2, ф‐β‐GAL and ф‐NGAL previously to infusion. B, Phenotype analysis was likewise analysed by FACS CANTO cytometry and BM‐фM2 and ф‐NGAL showed high expression of CD206 compared to ф‐β‐GAL. n = 3 in each group. ^a^ *P* ≤ 0.05 BM‐фM2 vs ф‐NGAL; ^b^ *P* ≤ 0.05 ф‐NGAL vs ф‐β‐GAL

Transduction of Φ‐βGAL macrophages showed slight mRNA expression of IL‐10, TNF‐α, NGAL and CD206 molecules compared to BM‐ΦM2 and Φ‐NGAL macrophages (Figure 6A). Furthermore, Φ‐βGAL displayed no expression of the alternative M2 phenotype (CD206) (Figure 6B), contrary to BM‐ΦM2 and Φ‐NGAL macrophages that yielded ≥97% of CD206 positive marker, which demonstrated their alternative M2 phenotype analysed by FACS cytometer (Figure 6B).

## 4 DISCUSSION

In this study, we demonstrated firstly that BM‐ΦM2 macrophage cell therapy did not exert any therapeutic effect on DKD and secondly that Φ‐NGAL macrophage cell therapy was associated with a therapeutic effect on DKD. The lack of therapeutic effect of the BM‐ΦM2 macrophage cell therapy is consistent with our previous findings in the CKD UUO model that demonstrates M2 switching to M1 in a pro‐inflammatory and pro‐fibrotic environment.^22^

Hyperglycaemia and the dysregulated metabolic milieu play a key role in DKD.^26^ However, in TD2M tight glucose control was not associated with improved renal and non‐renal outcome.^27^, ^28^, ^29^ Thus, although hyperglycaemia plays a major role in DKD initiation additional effector mechanisms should be involved in the progression of DKD. Among such mechanisms, inflammation is considered as a relevant pathogenic factor in the progression of DKD.^30^ Therefore, the reduction in inflammation has been associated with a significant reduction in the extracellular matrix accumulation in DKD.^31^

NGAL has been described as a marker and potential positive modulator of inflammation.^32^ NGAL display anti‐inflammatory properties in different diseases providing protective effects. One of the main acting pathways is by interfering the transcription of NF‐kB dependent genes such as TNF‐alpha and IL‐6.^33^ Recently, it has been described NGAL interaction in inflammation by controlling autophagy^34^ and inflammasome.^35^ Moreover, in the kidney, NGAL can indirectly reduce inflammation by blocking necrosis and by modulating apoptosis and more specifically by activating DAMP HMGB‐1, as demonstrate in the nephrotoxic serum nephritis.^36^ Hence, NGAL may also be one of the key potential modulators of macrophage phenotype stabilization and inflammation^37^, ^38^; thus, promoting renal epithelial cell integrity and renal recovery after injury.^24^ Our group has recently described^39^ that, in renal epithelial cells, Lcn‐2 acts via binding to its specific receptor and triggers downstream pathways of proliferation (activation of the PI3K/Akt‐pathway) and inhibition of PPARγ. Previous data from our group suggested that the infusion of Φ‐NGAL macrophages, as a result of their IL‐10 overexpression, was effective during the inflammatory phase of renal ischaemia and reperfusion injury.^40^ We have also recently reported that Φ‐NGAL macrophages have reduced cell plasticity and therefore preserve their anti‐inflammatory and anti‐fibrotic phenotype even when placed in a pro‐inflammatory and pro‐fibrotic environment.^22^

Taking into account this rationale we investigated whether Φ‐NGAL macrophage cell therapy could overcome the limitations of BM‐ΦM2 and halt the progression of DKD as infused Φ‐NGAL macrophages reach the diabetic kidney. TNF‐α is probably one of most relevant inflammatory cytokines involved in DKD. Several studies suggest that inhibition or altering TNF‐α may exert therapeutic effect on DKD^41^, ^42^ even in humans.^43^ In our study Φ‐NGAL macrophage cell therapy increased the anti‐inflammatory molecule IL‐10 and decreased the pro‐inflammatory molecule TNF‐α, suggesting that Φ‐NGAL macrophages could reduce inflammation in DKD. The anti‐inflammatory effect may have both a direct therapeutic effect on DKD and at the same time could contribute to M2 phenotype stabilization. The lower proportion of glomerular pro‐inflammatory M1 macrophages observed in the Φ‐NGAL group is in agreement with M2 phenotype stabilization. In fact, we have previously reported that the reduction in pro‐inflammatory cytokines increased the proportion of reparative and anti‐inflammatory M2 macrophages in diabetic glomeruli.^25^

In a seminal investigation published more than 20 years ago, Pagtalunan et al^44^ recognized podocyte loss as a relevant contributor to the progression of DKD. It has been described that podocyte number correlates with proteinuria and it is one of the best predictors of DKD.^45^ In addition to other pathogenic mechanisms as hyperglycaemia‐induced ROS generation,^26^ inflammation^46^ can contribute to podocyte loss by causing podocyte apoptosis and detachment. As podocytes are terminally differentiated cells that cannot be replaced, the remaining podocytes enlarge by cell hypertrophy and finally dedifferentiate to cell type that contribute to extracellular matrix accumulation. In our study, we found that only infusion of Φ‐NGAL macrophages exerted a protective effect on podocyte loss and was associated with a significant reduction in albuminuria. As this cell therapy was also the only one that could modulate the inflammatory response in DKD, it has been suggested that Φ‐NGAL macrophage cell therapy preserve podocyte number by means of reducing inflammation. Therefore, our results are in‐line with other studies suggesting that the reduction in podocyte damage may, in fact, reduce albuminuria and diabetes progression.^47^ Albuminuria is a biomarker of DKD and its severity is considered one of the most important predictors of DKD progression.^48^ As previously pointed out, the reduction in albuminuria in the Φ‐NGAL group may merely by a reflection of podocyte preservation. Nevertheless, albuminuria in pathological states such as DKD, can be also because attenuated tubular reabsorption of albumin.^49^ Megalin is a large glycoprotein that is highly expressed in proximal tubular epithelial cells.^50^ As we found that Φ‐NGAL macrophage cell therapy was associated with preserved megalin gene expression and consequently with tubular epithelial integrity, it is feasible that albumin tubular reabsorption could also play a role to reduce albuminuria in diabetic animals treated with Φ‐NGAL.

The fibrogenic cytokine transforming growth factor β (TGF‐β) is an important transcriptional regulator of cell transdifferentition promoting epithelial to mesenchymal transition and extracellular matrix accumulation. There is consistent data showing that renal TGF‐β expression increases in DKD^6^, ^51^ as it is induced by renin angiotensin system activation, metabolic dysregulation and hyperglycaemia. TGF‐β is considered as one of the most relevant pro‐fibrotic factors accounting for glomerulosclerosis and interstitial fibrosis in DKD as well as other chronic nephropathies.^52^, ^53^ The administration of anti‐ TGF‐β antibodies, antisense TGF‐β1 oligodeoxynucleotides or knocking off the downstream Smad3 gene prevent and/or reverse the hypertrophic and pro‐fibrotic effects of hyperglycaemia in DKD.^54^

Considering the potential benefit of M2 macrophage cell therapy on DKD it is important to note that M2 macrophages have anti‐inflammatory properties but are also associated with fibrosis as they can secrete TGF‐β.^19^, ^20^ Therefore, in our study we found that in mice treated either with BM‐ΦM2, Φ‐BM and Φ‐RAW infusion, the renal TGFβ‐1 as well as renal fibrosis were similar to that observed in diabetic non‐treated animals. In order to overcome this limitation, we treated the diabetic mice with a phenotypically stabilized M2 cell line expressing low TGF‐β by transducing NGAL. Interestingly, the Φ‐NGAL macrophage cell therapy effectively reduced the elevated renal TGF‐β1 as well as the fibrotic renal lesions that are the hallmark of DKD. This effect was dependent on NGAL transduction because the Φ‐RAW group showed similar renal TGFβ‐1 and fibrosis than the diabetic control group. The lack of therapeutic effect of BM‐ΦM2 may be because of M1 switching, TGF‐β1 secretion or both. Actually, in a previous study we demonstrated that both BM‐ΦM2 and Φ‐NGAL infused macrophages reach the damaged kidney although the BM‐ΦM2 switch significantly their phenotype to the inflammatory one M1.^22^ Also, Zheng et al^55^ in diabetic animals proved that macrophages from the cell therapy were located in spleen immediately after infusion, and then accumulated progressively both in kidney and pancreas.

The immunogenicity of those Φ‐RAW macrophages could be interpreted as a potential limitation in our study. Φ‐RAW macrophages derive from monocytic leukaemic cell of BALB/c that carries H‐2d, and C57Bl/6j carries H‐2b. Therefore, adoptive transfer of cells to mice with different H‐2 antigen is possibly immunogenic. However, previous studies from our group and others,^22^, ^23^, ^56^, ^57^ suggested that infusing these cells in C57Bl/6j was not immunogenic. Consistent with these results, we did not detect any effect associated with the potential activation of an effector alloimmune response against the infused cells in our diabetic mice model.

In summary, our study demonstrates that Φ‐NGAL macrophage cell therapy has a therapeutic effect on DKD in diabetic mice probably by modulation of the renal inflammatory response caused by the diabetic milieu. Our study provides evidence about the limitations of macrophage cell therapy in kidney diseases and shows a potential strategy to overcome the inherent M2 plasticity and TGF‐β1 overexpression by Φ‐NGAL transduction. Thus, our study paves the way to new opportunities in DKD treatment.

## CONFLICT OF INTEREST

All the authors declared no competing interests.

## AUTHOR CONTRIBUTION

JMC and RG developed the concept and designed the research; RG performed experiments and RG, AS and JMC analysed the data. JMC and RG interpreted the data and wrote the manuscript. RG generated all figures and JMC supervised the project. All authors discussed the results and implications and commented on the manuscript at all stages. Josep Maria Cruzado is the guarantor of this work and, as such, had full access to all of the data in the study and takes responsibility for the integrity of the data and the accuracy of the data analysis.
