# Supplementary figures and images for: Exploring macrophage cell therapy on Diabetic Kidney Disease
Source: J Cell Mol Med. 2018 Nov 8;23(2):841–51. doi: 10.1111/jcmm.13983 (PMC6349346; doi:10.1111/jcmm.13983)

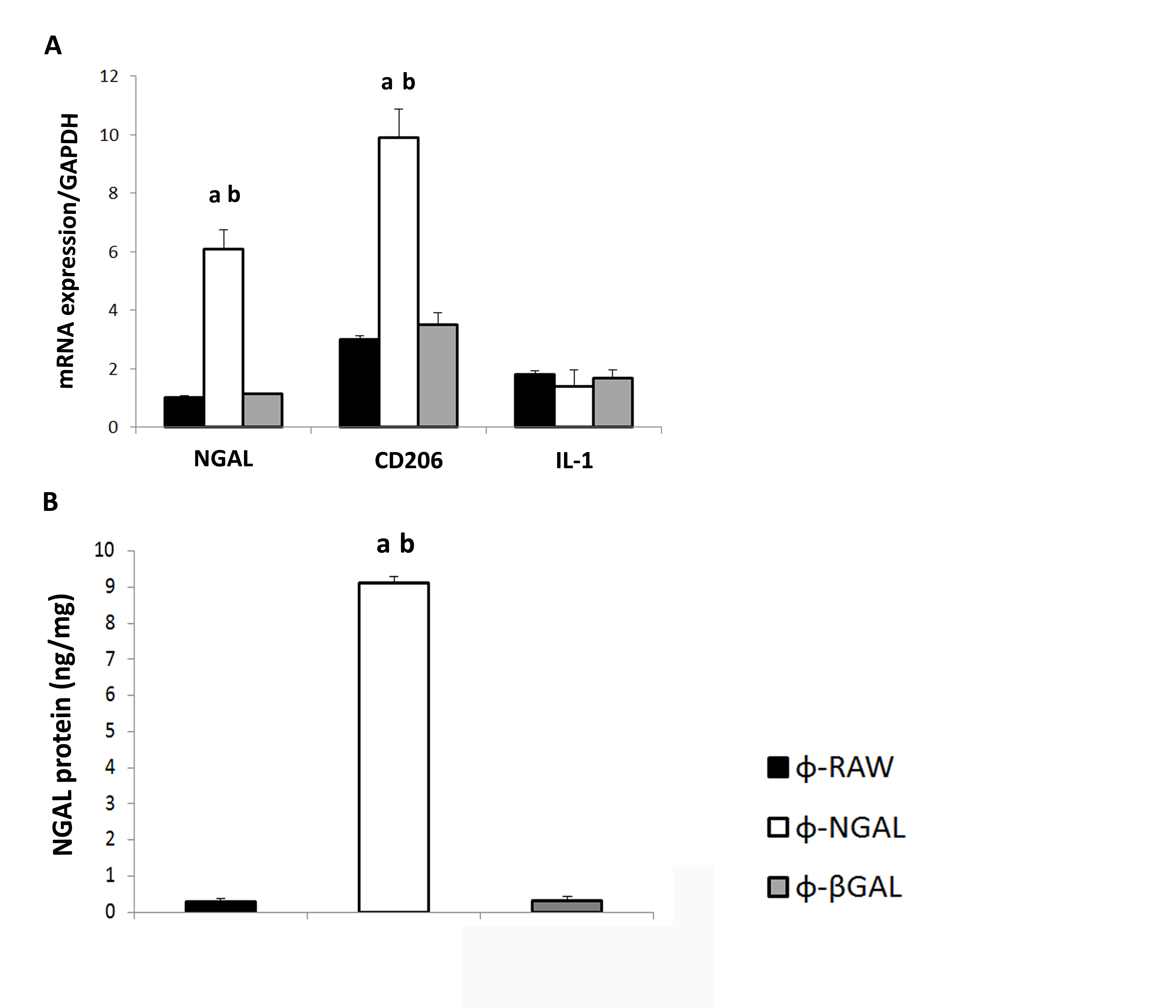

Supplement: Supplementary file 1 [file JCMM-23-841-s001.tif]

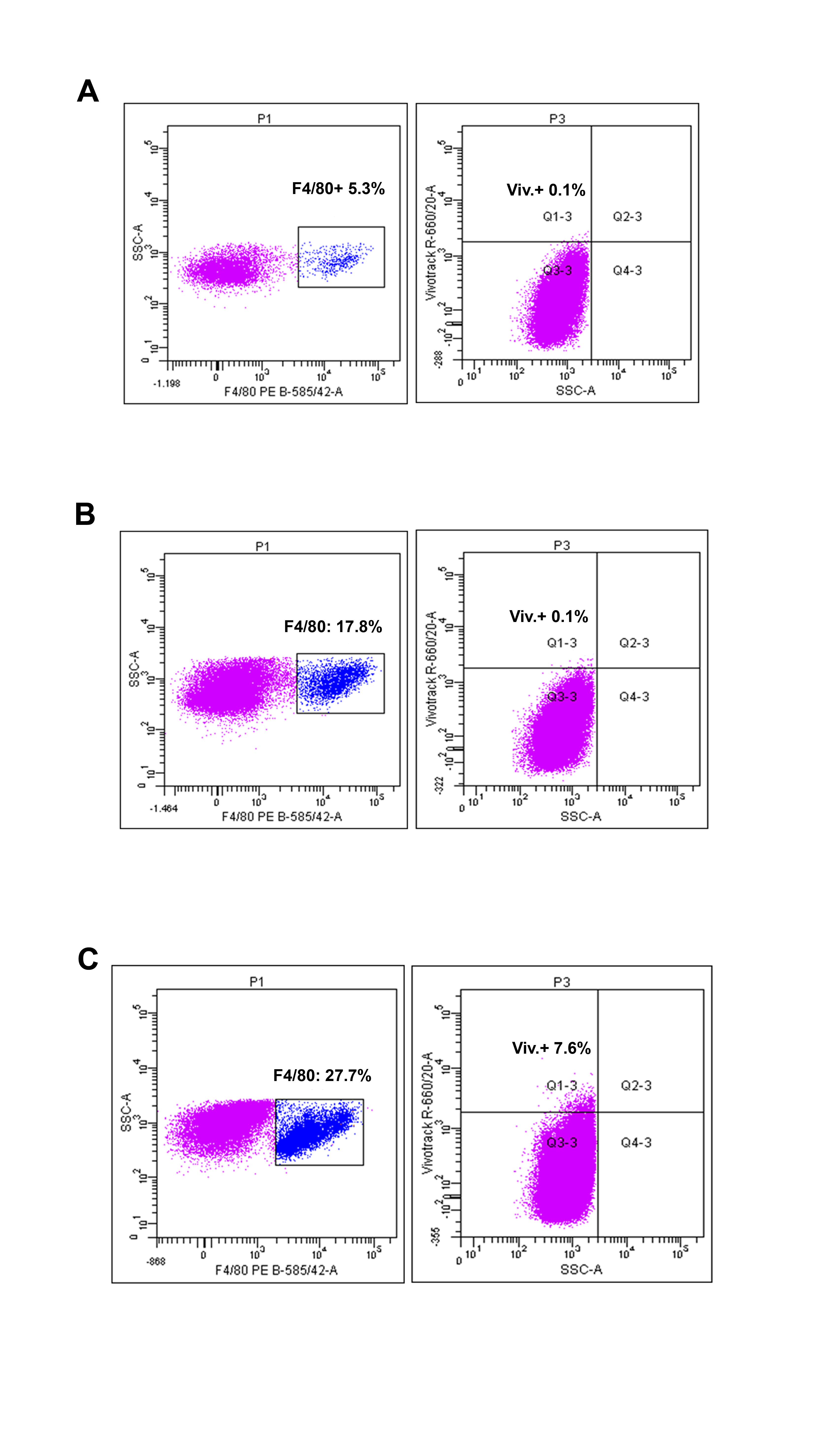

Supplement: Supplementary file 2 [file JCMM-23-841-s002.tif]
